# Construction of Nilpotent and Solvable Lie Algebra in Picture Fuzzy Environment

**DOI:** 10.1007/s44196-023-00213-w

**Published:** 2023-03-21

**Authors:** Sajida Kousar, Sidra Arshad, Nasreen Kausar, Tzung-Pei Hong

**Affiliations:** 1 Department of Mathematics and Statistics, International Islamic University, Sector H-10, Islamabad, 04436 Pakistan; 2 Department of Mathematics, Faculty of Arts and Sciences, Yildiz Technical University, Esenler, 34220 Istanbul, Turkey; 3 Department of Computer Science and Information Engineering, National University of Kaohsiung, Kaohsiung, 811 Taiwan; 4 Department of Computer Science and Engineering, National Sun Yat-sen University, Kaohsiung, 804 Taiwan

**Keywords:** Picture fuzzy set, Lie algebra, Picture fuzzy Lie algebra, Nilpotent and solvable picture fuzzy Lie algebra

## Abstract

The picture fuzzy set was introduced by Coung. It is a generalization of the intuitionistic fuzzy set, giving the notion of neutral membership degrees along with the positive and negative ones. Lie groups and Lie algebras have become indispensable for a lot of fields in mathematical and intellectual physics. In 1872, Lie began his work in the field of continuous transformation groups, later named after him as Lie groups. These have become a fundamental body of interest in themselves. In this paper, the authors presented the notion of the picture fuzzy Lie algebra, picture fuzzy Lie sub-algebra, ideal, and homomorphism. Derived and lower central series of picture fuzzy Lie ideals are constructed to define and analyse solvable and nilpotent picture fuzzy Lie ideals.

## Introduction

The concept of Lie algebra was first studied by Sophus Lie [1] in the 1870 s as continuous transformation groups (now termed as Lie groups). This later lead to the study of Lie algebras. Over the years, many researchers have worked on Lie algebra and established several structural concepts aligned with groups, rings, and other algebraic structures. Lie groups, Lie algebras and Lie similarities are essential factors for advancement in theoretical and experimental physics.

The probability theory was once believed to be an ideal tool for handling imprecise and vague data especially in quantum mechanics [2]. The relative frequencies of obtaining various results in certain experiments on quantum systems are interpreted as probabilities and fail to fulfill the numerical constraints levied by the classical probability theory. These situations are usually connected with the violation of Bell’s inequalities [3] and strongly indicate the necessity of modifying the probability calculus used in quantum mechanics. The fuzzy set theory offers not only an influential, significant and powerful role in the depiction of imprecision and uncertainties, but also caters the subjectivity and vagueness of natural languages. To handle with uncertainty and imprecision, the notion of fuzzy sets was formerly proposed by Zadeh [4] in 1965. The concept can be made understood by a simple example. Let *C* be the set of countries affected by the COVID pandemic and *x* be a country in *C* with a total population *p*(*x*). If *q*(*x*) is the number of people tested positive for COVID-19 recorded by the government’s authorized database, then *A* is the fuzzy set of *C* with COVID positive, having membership grade $\zeta _{A}(x) = \frac{q(x)}{p(x)}$. The fraction of the population not tested positive is then $1- \zeta _A(X)$, but many people may get a false negative or false positive report. This may change the truth/positive degrees $\zeta _A(x)$, and falsity/negative degrees 1 - $\zeta _A(x)$, and ultimately the probabilistic concept of occurrence and non-occurrence of an event does not support this situation. Keeping these anomalies under consideration, Attanasov [5] laid the notion of the intuitionistic fuzzy set -a generalization of fuzzy set- in 1983. Thus according to the intuitionistic fuzzy set, if $\xi (x)$ (termed as a non-membership grade) is the fraction of the population tested negative for COVID, then $0 \le \zeta _A(x) + \xi _A(x) \le 1$. It is widely observed that several people, affected by the corona virus either showed no symptoms or refused to get tested due to illiteracy, rumors or other social factors. This leads toward the grade of neutral membership $\eta _A(x)$. Thus four kinds of grades can be assigned to the population, namely “positive membership” (people who are tested positive), “neutral membership” (people who refused to get tested), “negative membership” (people who are tested negative) and “refusal of membership” (people who were unable to approach test centers) defined as 1 - $(\zeta A(x) + \eta A(x) + \xi A(x))$. This concept is formally defined by Cuong [6], who established the theory of picture fuzzy sets and extended the fundamental set operations to these sets. As it is known that the algebraic structures are essential in many fields of mathematics and other sciences, this vitality was a motivation to study these structures in a fuzzy environment. This milestone was initially achieved by Rosenfeld [7] in 1971, who characterized the concept of fuzzy subgroups. Later in 1996, Yehia [8] extended the branch of Lie algebra to fuzzy Lie algebra. He developed the concept of fuzzy Lie ideals and studied their properties. Some of their properties were also studied by Akram [9–13], where he defined the level sets and characterized the fuzzy Lie ideals of Artinian and Noetherian Lie Algebras. He also defined the interval-valued $(\epsilon , q, \epsilon \vee q)$-fuzzy Lie algebras [14], where $\epsilon , q$, and $\epsilon \vee q$ symbols belong to quasi-coincident and or quasi-coincident with a fuzzy set, respectively. After defining the fuzzy Lie sub-algebras in 1996, Yehia [15], in 2001, illustrated the Killing form in a fuzzy context and proposed the fuzzification of the adjoint representation of Lie algebras. He then established a relation between this representation and nilpotent fuzzy Lie ideals. Eghdami et al. [16] studied fuzzy roughness in Lie algebras and put forward the concept of reference points. Zadeh and Ameri [17], developed the notion of characterizing fuzzy subgroups as fuzzy Lie rings. The existence and uniqueness of nilpotent radicals were proved by Ferriera and Marietto [18]. Besides, Chen [19] investigated some essential properties of the intuitionistic fuzzy quotient Lie super algebra. Antony [20] by considering $\alpha , \beta \in \{\in , q, \in \vee q\}$ discovered the $(\alpha , \beta )$-fuzzy Lie algebra over an $(\alpha , \beta )$-fuzzy field and peculiarized the $(\epsilon , \epsilon \vee q)$-fuzzy Lie algebra over an $(\epsilon , \epsilon \vee q)$-fuzzy field. These are the pieces of work done over fuzzy sets where a membership function takes the value from [0, 1]. Here we must not miss the remarkable work done by Shaqaqah [21], who took the fuzzy Lie algebras to a higher level. He studied the Lie algebras in the complex fuzzy environment, where a membership function takes values in the unit circle in the complex plane.

Applications of Lie algebra are extensively found in physics and robotics. The outcomes of these applications have proven to be of great importance to humankind. Considering the utmost importance, many scientists have worked on the applications of Lie algebra. An example is the angular momentum operator in quantum mechanics, which is among the several related operators analogical to the classical angular momentum. The angular momentum operator is an essential tool in the theory of atomic and molecular physics and becomes inevitable in many other quantum problems based on rotational symmetry. In both classical and quantum mechanical systems, angular momentum is one of the three fundamental properties of motion. There are several angular momentum operators: orbital angular momentum $L = r \times p$ where *r* is the quantum position operator and *p* is the quantum momentum operator, spin angular momentum $S = (S_{x}, S_{y}, S_{z})$ indicating spinning of a particle around an axis and total angular momentum $J = L + S$. However, the term of the angular momentum operator can refer to either the total or the orbital angular momentum. The components of the orbital angular momentum satisfy the following relations:

$$\begin{aligned} {}[L_{x},L_{y}] = \iota \hbar L_z, \,[L_y,L_z] = \iota \hbar L_x, \,[L_z,L_x] = \iota \hbar L_y \end{aligned}$$

where $\iota = \sqrt{-1}$ and [, ] denotes the Lie bracket with the following calculation:

$$\begin{aligned} {}[X, Y ] = XY - YX. \end{aligned}$$

This implies that *L* has the mathematical structure of a Lie algebra.

Other scientists have worked on tensor products, rigid body controllers, mobility of parallel platforms, 3-D laser scanners, control algorithms of a non-holonomic manipulator, and jointly apportioned poses [22–27]. Real-world problems are not ideal. Rather than being black and white, persistently have gray areas. In other words, practical circumstances are always uncertain and not precise. Previously, Lie algebra was studied in the fuzzy and intuitionist fuzzy environments and can handle only truth and falsity without considering the hesitancy error occurring in terms of uncertainty or indeterminacy. The importance of Lie algebra in experimental physics and quantum mechanics, and the uncertain and imprecise outcomes of experiments where the probability theory, fuzzy set, and intuitionistic fuzzy set failed to satisfy the fundamental laws of the said fields, are evident enough for further modification in the set/logic under consideration. Clearly, the structures of fuzzy Lie algebra and intuitionistic fuzzy Lie algebra may not completely address the anomalies and ambiguities occurring in real-life scenarios, keeping in mind the imprecision of fuzzy and intuitionistic fuzzy sets. As mentioned earlier, the picture fuzzy set is a generalization of both the fuzzy and intuitionistic fuzzy sets and is highly efficient in dealing with the ambiguities in a large scale of data. The efficacy of picture fuzzy sets motivated the author to work on the notion of picture fuzzy Lie algebra which has been presented in this article. This provides another framework to the scientists to analyze the expectations and outcomes of their scientific discoveries. Following the notion of fuzzy Lie algebra, structural properties like sub-algebra, ideal, and homomorphism of Lie algebra are discussed under picture fuzzy environment, glorified with algebraic results and justified by providing examples. This is processed by using the definitions of Lie algebra, ideal, and homomorphism, reviewed in Sect. 2. The series of Lie algebras are then used to construct the series of picture fuzzy Lie ideals, which are in turn, used to study the nilpotent and solvable Lie algebra in the picture fuzzy environment.

## Basic Concepts

In this section, we will briefly study the concepts that will be used in this research.

### Definition 1

[1] A Lie algebra *L* is a vector space over the field ${\mathbb {F}}$ together with a bi-linear map -$[\cdot ,\cdot ]: L \times L \longrightarrow L$- known as the Lie bracket satisfying alternativity, the Jacobi identity, and anti-commutativity i.e. $\forall \;u,\; v,\; w,\; x_{1}$, $x_{2},\; y_{1},\; y_{2} \in L$ and $c,\; d \in {\mathbb {F}}$:

$$\begin{aligned} {}[u,u]& =  0;\\ {}[u,[v,w]] + [v,[w,u]] + [w,[u,v]]& =  0;\\ {}[u,v]& =  -[v,u], \text { and} \end{aligned}$$

for bi-linearity $[cx_{1}+dx_{2},v] = c[x_{1},v] + d[x_{2},v]$ and $[u,cy_{1}+dy_{2}] = c[u,y_{1}] + d[u,y_{2}]$.

### Definition 2

[1] Let $L_{1}$ and $L_{2}$ be two Lie algebras over the same field ${\mathbb {F}}$. A map

$$\begin{aligned} \theta : L_{1} \longrightarrow L_{2} \end{aligned}$$

is called Lie homomorphism if the following two conditions are satisfied:

1. $\theta (ax+by) = a\theta (x) + b\theta (y)$ i.e. $\theta$ is a linear transformation;
2. $\theta ([x,y]_{L_{1}}) = [\theta (x), \theta (y)]_{L_{2}}$ i.e. $\theta$ preserves the Lie bracket.

The intuitionistic fuzzy set [5] is a generalization of the fuzzy set. While the fuzzy set is described only in terms of the membership function, the intuitionistic fuzzy set gives information about the degree of membership $\zeta _{A}(u)$ as well as the degree of non-membership $\xi _{A}(u)$ of a parameter simultaneously. Both the functions (membership and non-membership) assign to each element in the universe of discourse a value within the closed interval [0, 1] and have an additional limitation:

$$\begin{aligned} 0 \le \zeta _{A}(u) + \xi _{A}(u) \le 1, \end{aligned}$$

where $\zeta _{A}: X \longrightarrow [0,1]$ and $\xi _{A}: X \longrightarrow [0,1]$ and

$$\begin{aligned} A = \{\left\langle u,\zeta _{A}(u), \xi _{A}(u) \right\rangle \,:\; u \in X \}. \end{aligned}$$

The picture fuzzy set was introduced by Coung [6] and is a direct extension of the fuzzy set and intuitionistic fuzzy set. It integrates the degrees of membership and non-membership (as the degrees of positive and negative memberships of a parameter), and introduces the degree of the neutral membership. It is defined as follows.

### Definition 3

[6] Let *A* be a picture fuzzy set on a universal set *L*, and $\zeta _{A}(u)$, $\eta _{A}(u)$ and $\xi _{A}(u) \in [0,1]$ be the degree of positive, neutral and negative membership of $u \in L$ respectively. Then

$$\begin{aligned} A= \{\left\langle u, \zeta _{A}(u),\eta _{A}(u), \xi _{A}(u)\right\rangle \,:\,u \in L \},\ \end{aligned}$$

and

$$\begin{aligned} \forall \; u \in L, \quad 0 \le \zeta _{A}(u)+\eta _{A}(u)+\xi _{A}(u) \le 1. \end{aligned}$$

$(1-(\zeta _{A}(u)+\eta _{A}(u)+\xi _{A}(u)))$ can now be called the degree of refusal of *u* in *L*.

Cuong [6] also extended the basic set operations to picture fuzzy sets. For two picture fuzzy sets, *A* and *B*, on a Lie algebra *L*, Cuong defined $A \subseteq B, \; A=B, \; A \cap B, \; A \cup B, \text {and}\; co(A)$.

Before putting forward our main results, it may be useful to give a brief background of the fuzzy Lie algebra.

### Definition 4

[8] Let *A* be the fuzzy set of *L*(a Lie algebra). We say that *A* is a fuzzy Lie sub-algebra if

1. $$\zeta _A(u_{1}+v_{1}) \ge \min \{\zeta _A(u_{1}), \zeta _A(v_{1})\}$$,
2. $$\zeta _A(cu) \ge \zeta _A(u)$$, and
3. $$\zeta _A([u_{1},v_{1}]) \ge \min \{\zeta _A(u_{1}), \zeta _A(v_{1})\}$$,

for every $u,u_{1}, v_{1} \in L$ and every $c\in {\mathbb {F}}$.

If the third condition is replaced by

$$\begin{aligned} \zeta _A([u_{1},v_{1}]) \ge \max \{\zeta _A(u_{1}), \zeta _A(v_{1})\}, \end{aligned}$$

then *A* is known as the fuzzy Lie ideal.

### Definition 5

[8] Let $J^{L_{1}}$ and $J^{L_{2}}$ be the collections of all the fuzzy Lie algebras of $L_{1}$ and $L_{2}$ respectively. Let $\omega : L_{1} \longrightarrow L_{2}$ be a Lie homomorphism and *A* be a fuzzy Lie algebra of $L_1$. Then $\omega (A)$ is a fuzzy Lie algebra of $L_2$ with membership function defined as:

$$\begin{aligned} \omega (\zeta _A)(r)= \sup \{\zeta _A(s) \,:\, s\in \omega ^{-1}(r)\}. \end{aligned}$$

The extension of $\omega$ from $J^{L_{1}} \longrightarrow J^{L_{2}}$ is a homomorphism of the fuzzy sets over Lie algebras.

### Definition 6

[8] Consider a fuzzy Lie ideal *S* of $L\; (\text {Lie algebra})$. The derived series of *S* with the *k*th derived fuzzy Lie ideal $S^{k}$ is

$$\begin{aligned} S^{0} \supseteq S^{1} \supseteq \cdots \supseteq S^{k} \supseteq \ldots \end{aligned}$$

where

$$\begin{aligned} S^{0}=S,\quad S^{1}= [S^{0}, S^{0}],\quad \ldots ,\quad S^{k}= [S^{k-1},\, S^{k-1}]. \end{aligned}$$

In the derived series, if $S^{k}= 0$, then we say that *S* is solvable.

### Definition 7

[10] For a fuzzy Lie ideal *N* of *L* (a Lie algebra) given below is the descending central series of *N*

$$\begin{aligned} N_{0} \supseteq N_{1} \supseteq \cdots \supseteq N_{k} \supseteq \ldots \end{aligned}$$

where

$$\begin{aligned} N_{0}=N,\quad N_{1}= [N_{0}, N_{0}],\quad \ldots ,\quad N_{k}= [N_{k-1}, N_{k-1}]. \end{aligned}$$

*N* is known as nilpotent if, in the descending central series, $N_{k}= 0_{\sim }$, where $0_{\sim }$ is the fuzzy Lie ideal with $\zeta _0(u)=0$ for all $u\in L$.

## Main Results

Before defining the picture fuzzy Lie sub-algebra, the level sets and the operations of picture fuzzy sets shall first be defined.

### Definition 8

Let $\varepsilon , \varsigma , \tau \in [0,1]$. Then the collections

$$\begin{aligned} U(\zeta _{A}, \varepsilon )=\{x\in L:\; \zeta _{A}(x) \ge \varepsilon \}\; \text {and} \;U(\eta _{A}, \varsigma ) = \{x \in L:\;\eta _{A}(x) \ge \varsigma \} \end{aligned}$$

are the upper-level sets of $\zeta _{A}$ and $\eta _{A}$ respectively, and the collection

$$\begin{aligned} L(\xi _{A}, \tau )= \{x\in L:\; \xi _{A}(x) \le \tau \} \end{aligned}$$

is the lower level set of $\xi _{A}$.

### Definition 9

Consider a picture fuzzy Set $A= (\zeta _{A},\eta _{A},\xi _{A})$ on *L* and $\varepsilon ,\; \varsigma ,\; \tau \; \in [0,1]$, with $\varepsilon +\varsigma +\tau \le 1$

1. An $(\varepsilon , \varsigma , \tau )$-level subset of *A* is defined as
  $$\begin{aligned} {\mathcal {A}}^{(\varepsilon , \varsigma , \tau )}= \{u \in L\;:\; \varepsilon \le \zeta _{A}(u), \beta \le \eta _{A}(u), \xi _{A}(u) \le \gamma \}. \end{aligned}$$
  Whereas, an $(\varepsilon , \varsigma , \tau )$-level subset of *A* is the set of all $(\varepsilon , \varsigma , \tau ) \in \zeta _{A}(L)\times \eta _{A}(L) \times \xi _{A}(L)$ so that $\varepsilon + \varsigma + \tau \le 1$.
2. A strong $(\varepsilon , \varsigma , \tau )$-level subset of *A* is given as
  $$\begin{aligned} A_{(\varepsilon , \varsigma , \tau )}= \{u\in L:\;\varepsilon< \zeta _{A}(u),\; \varsigma< \eta _{A}(u),\; \xi _{A}(u)<\tau \}. \end{aligned}$$

It must be noted that

$$\begin{aligned}{} & {} {\mathcal {A}}^{(\varepsilon , \varsigma , \tau )}=\{u\in L:\; \zeta _{A}(u) \ge \varepsilon , \; \eta _{A}(u) \ge \varsigma ,\; \xi _{A}(u) \le \tau \} \\{} & {} = U(\zeta _{A}, \varepsilon )\cap U(\eta _{A}, \varsigma ) \cap L(\xi _{A},\tau ). \end{aligned}$$

### Definition 10

Consider two picture fuzzy Sets $P=(\zeta _{P}, \eta _{P}, \xi _{P})$ and $Q=(\zeta _{Q}, \eta _{Q}, \xi _{Q})$ of *L* (Lie algebra). Then

$$\begin{aligned} {}[PQ]=\{\left\langle u, [\zeta _{P}\zeta _{Q}](u),[\eta _{P}\eta _{Q}](u), [\xi _{P}\xi _{Q}](u)\right\rangle :\; u \in L\} \end{aligned}$$

is the picture fuzzy formed by their product with degrees defined as:

$$\begin{aligned}{}[\zeta _{P}\zeta _{Q}](u)&= \left\{ \begin{array}{c c} \sup \{\min \{\zeta _{P}(v), \zeta _{Q}(w)\}\}, &{} \hbox { if } u = vw; \\ 0, &{} \hbox { if } u \ne vw. \end{array} \right. \\ [\eta _{P}\eta _{Q}](u)&= \left\{ \begin{array}{c c} \sup \{\min \{\eta _{P}(v), \eta _{Q}(w)\}\}, &{} \hbox { if } u = vw; \\ 0, &{} \hbox { if } u \ne vw. \end{array} \right. \\ [\xi _{P}\xi _{Q}](u)&= \left\{ \begin{array}{c c} \inf \{\max \{\xi _{P}(v), \xi _{Q}(w)\}\}, &{} \hbox { if } u = vw; \\ 0, &{} \hbox { if } u \ne vw. \end{array} \right. \end{aligned}$$

The picture fuzzy set

$$\begin{aligned} \left\langle \left\langle PQ\right\rangle \right\rangle = \{\left\langle u, \left\langle \left\langle \zeta _{P}\zeta _{Q} \right\rangle \right\rangle (u), \left\langle \left\langle \eta _{P} \eta _{Q}\right\rangle \right\rangle (u), \left\langle \left\langle \xi _{P}\xi _{Q} \right\rangle \right\rangle (u) \right\rangle :\; u \in L\} \end{aligned}$$

generated by *P* and *Q* is formalized as follows:

$$\begin{aligned} \left\langle \left\langle \zeta _{P}\zeta _{Q}\right\rangle \right\rangle (u)&= \left\{ \begin{array}{c c} \sup \{\min _{i \in {\mathbb {N}}}\{\min \{\zeta _{P}(v_{i}), \zeta _{Q}(w_{i})\}\}\}, &{} \hbox { if } u=\sum _{i=1}^{n}v_{i}w_{i}; \\ 0, &{} \hbox { if } u\ne \sum _{i=1}^{n}v_{i}w_{i}. \end{array} \right. \\ \left\langle \left\langle \eta _{P}\zeta _{Q}\right\rangle \right\rangle (u)&= \left\{ \begin{array}{c c} \sup \{\min _{i \in {\mathbb {N}}}\{\min \{\eta _{P}(v_{i}), \eta _{Q}(w_{i})\}\}\}, &{} \hbox { if } u=\sum _{i=1}^{n}v_{i}w_{i}; \\ 0, &{} \hbox { if } u\ne \sum _{i=1}^{n}v_{i}w_{i}. \end{array} \right. \\ \left\langle \left\langle \xi _{P}\zeta _{Q}\right\rangle \right\rangle (u)&= \left\{ \begin{array}{c c} \inf \{\max _{i \in {\mathbb {N}}}\{\max \{\xi _{P}(v_{i}), \xi _{Q}(w_{i})\}\}\}, &{} \hbox { if } u=\sum _{i=1}^{n}v_{i}w_{i}; \\ 0, &{} \hbox { if } u\ne \sum _{i=1}^{n}v_{i}w_{i}. \end{array} \right. \end{aligned}$$

### Definition 11

The Lie product of $P=(\zeta _{P}, \eta _{P}, \xi _{P})$ and $Q=(\zeta _{Q}, \eta _{Q}, \xi _{Q})$), i.e.,

$$\begin{aligned}{}[P,Q] = (\zeta _{[P,Q]}, \eta _{[P,Q]}, \xi _{[P,Q]}) \end{aligned}$$

can now be defined as follows:

$$\begin{aligned} \zeta _{[P,Q]}(u)&= \left\{ \begin{array}{c c} \sup \{\min _{i \in {\mathbb {N}}}\{\min \{\zeta _{P}(v_{i}), \zeta _{Q}(w_{i})\}\}\}, &{} \hbox { if } u=\sum _{i=1}^{n}[v_{i}, w_{i}]; \\ 0, &{} \hbox { if } u\ne \sum _{i=1}^{n}[v_{i}, w_{i}]. \end{array} \right. \\ \eta _{[P,Q]}(u)&= \left\{ \begin{array}{c c} \sup \{\min _{i \in {\mathbb {N}}}\{\min \{\eta _{P}(v_{i}), \eta _{Q}(w_{i})\}\}\}, &{} \hbox { if } u=\sum _{i=1}^{n}[v_{i}, w_{i}]; \\ 0, &{} \hbox { if } u\ne \sum _{i=1}^{n}[v_{i}, w_{i}]. \end{array} \right. \\ \xi _{[P,Q]}(u)&= \left\{ \begin{array}{c c} \inf \{\max _{i \in {\mathbb {N}}}\{\max \{\xi _{P}(v_{i}), \xi _{Q}(w_{i})\}\}\}, &{} \hbox { if } u=\sum _{i=1}^{n}[v_{i}, w_{i}]; \\ 0, &{} \hbox { if } u\ne \sum _{i=1}^{n}[v_{i}, w_{i}]. \end{array} \right. \end{aligned}$$

We can now define and discuss the picture fuzzy Lie sub-algebra and ideal, which are of main interest.

### Definition 12

If a picture fuzzy set $A=(\zeta _{A}, \eta _{A}, \xi _{A})$ on *L* (a Lie algebra) satisfies the following given axioms:

1. $$\zeta _{A}(x_{1}+x_{2}) \ge \min \{\zeta _{A}(x_{1}), \zeta _{A}(x_{2})\}; \; \eta _{A}(x_{1}+x_{2}) \ge \min \{\eta _{A}(x_{1}), \eta _{A}(x_{2})\}; \xi _{A}(x_{1}+x_{2}) \le \max \{\xi _{A}(x_{1}), \xi _{A}(x_{2})\},$$
2. $$\zeta _{A}(cx_{1}) \ge \zeta _{A}(x_{1}); \; \eta _{A}(cx_{1}) \ge \eta _{A}(x_{1}); \; \xi _{A}(cx_{1}) \le \xi _{A}(x_{1}),$$
3. $$\zeta _{A}([x_{1},x_{2}]) \ge \min \{\zeta _{A}(x_{1}), \zeta _{A}(x_{2})\}; \; \eta _{A}([x_{1},x_{2}]) \ge \min \{\eta _{A}(x_{1}), \eta _{A}(x_{2})\}; \xi _{A}([x_{1},x_{2}]) \le \max \{\xi _{A}(x_{1}), \xi _{A}(x_{2})\}$$.

For every $x_{1}, x_{2} \in L$, then it is termed as picture fuzzy Lie sub-algebra.

### Example 1

Let $${\mathbb {F}}= {\mathbb {R}}$$ and $$L = {\mathbb {R}}^{3}= \{(\ell _{1},\ell _{2},\ell _3) \;:\; \ell _{1},\ell _{2},\ell _{3} \in {\mathbb {R}}\}$$,

where, $[\cdot , \cdot ]: {\mathbb {R}}^{3} \times {\mathbb {R}}^{3} \longrightarrow {\mathbb {R}}^{3}$ is defined as the cross product on ${\mathbb {R}}^{3}$, i.e., $[u,v]= u \times v$. We define a picture fuzzy set $A=(\zeta _{A}, \eta _{A}, \xi _{A})$ as

$$\begin{aligned} \zeta _{A}(\ell _{1},\ell _{2},\ell _{3})&= \left\{ \begin{array}{c c} 0, &{} \hbox { if } \ell _{1}=\ell _{2}=\ell _{3}=0; \\ 0.8, &{} \hbox { if } \ell _{1} \ne 0, \ell _{2}=\ell _{3}=0; \\ 1, &{} \hbox { otherwise }. \end{array} \right. \\ \eta _{A}(\ell _{1},\ell _{2},\ell _{3})&= \left\{ \begin{array}{c c} 0.1, &{} \hbox { if } \ell _{1}=\ell _{2}=\ell _{3}=0; \\ 0, &{} \hbox { otherwise }. \end{array} \right. \\ \xi _{A}(\ell _{1},\ell _{2},\ell _{3})&= \left\{ \begin{array}{c c} 1, &{} \hbox { if } \ell _{1}=\ell _{2}=\ell _{3}=0; \\ 0.5, &{} \hbox { if } \ell _{1} \ne 0, \ell _{2}=\ell _{3}=0; \\ 0, &{} \hbox { otherwise }. \end{array} \right. \end{aligned}$$

Clearly, $A= (\zeta _{A}, \eta _{A}, \xi _{A})$ is a picture fuzzy Lie sub-algebra.

### Proposition 1

For any two picture fuzzy Lie sub-algebras *P* and *Q*, $\left\langle \left\langle PQ\right\rangle \right\rangle$ is a picture fuzzy Lie sub-algebra, but [*PQ*] may not be a picture fuzzy Lie sub-algebra.

By replacing the third axiom of the picture fuzzy Lie sub-algebra as given below, the picture fuzzy Lie ideal can be defined.

### Definition 13

Let $K = (\zeta _{K},\eta _{K},\xi _{K})$ be the picture fuzzy set of *L*. If *K* satisfies the following axioms:

1. $$\zeta _{K}(x_{1}+y_{1}) \ge \min \{\zeta _{K}(x_{1}),\zeta _{K}(y_{1})\}$$, $$\eta _{K}(x_{1}+y_{1}) \ge \min \{\eta _{K}(x_{1}),\eta _{K}(y_{1})\}$$, $$\xi _{K}(x_{1}+y_{1})\le \max \{\xi _{K}(x_{1}), \xi _{K}(y_{1})\}$$
2. $$\zeta _{K}(\alpha x_{1}) \ge \zeta _{K}(x_{1})$$, $$\eta _{K}(\alpha x_{1}) \ge \eta _{K}(x_{1})$$, $$\xi _{K}(\alpha x_{1}) \le \xi _{K}(x_{1})$$
3. If $\zeta _{K}([x_{1},y_{1}]) \ge \zeta _{K}(x_{1})$, $\eta _{K}([x_{1},y_{1}]) \ge \eta _{K}(x_{1})$, $\xi _{K}([x_{1},y_{1}]) \le \xi _{K}(x_{1})$, then *K* is a left ideal. If $\zeta _{K}([x_{1},y_{1}]) \ge \zeta _{K}(y_{1})$, $\eta _{K}([x_{1},y_{1}]) \ge \eta _{K}(y_{1})$, $\xi _{K}([x_{1},y_{1}]) \le \xi _{K}(y_{1})$, then *K* is a right ideal. If $\zeta _{K}([x_{1},y_{1}]) \ge \max \{\zeta _{K}(x_{1}), \zeta _{K}(y_{1})\}$, $\eta _{K}([x_{1},y_{1}]) \ge \max \{\eta _{K}(x_{1}), \eta _{K}(y_{1})\}$, $\xi _{K}([x_{1},y_{1}]) \le \min \{\xi _{K}(x_{1}), \xi _{K}(y_{1})\}$, then *K* is a two-sided ideal.

### Example 2

Consider the two-dimensional real vector space

$$\begin{aligned} {\mathbb {R}}^{2}=\{(u_{1},u_{2}): u_{1}, u_{2} \in {\mathbb {R}}\}. \end{aligned}$$

Then ${\mathbb {R}}^{2}$ is a Lie algebra corresponding to the Lie bracket $[\cdot , \cdot ]$ provided as the usual cross product. Consider a picture fuzzy set $A=(\zeta _{A},\eta _{A},\xi _{A})$ defined below:

$$\begin{aligned} \zeta _{A}(\ell _{1},\ell _{2})= \left\{ \begin{array}{c c} 0.5 &{} \ell _{1}=\ell _{2}=0 \\ 0.3 &{} \textrm{otherwise} \end{array} \right. , \quad \eta _{A}(\ell _{1},\ell _{2})= \left\{ \begin{array}{c c} 0.3 &{} \ell _{1}=\ell _{2}=0 \\ 0.1 &{} \textrm{otherwise} \end{array} \right. , \\ \xi _{A}(\ell _{1},\ell _{2})= \left\{ \begin{array}{c c} 0.3 &{} \ell _{1}=\ell _{2}=0 \\ 0.5 &{} \textrm{otherwise} \end{array} \right. . \end{aligned}$$

Then the set *A* is clearly, a picture fuzzy right (left/two-sided) Lie ideal of the Lie algebra ${\mathbb {R}}^{2}$.

### Remark 1

A picture fuzzy right (left/two-sided) Lie ideal is a picture fuzzy Lie sub-algebra, but the contrary may or may not be true.

### Lemma 1

If $A= (\zeta _{A}, \eta _{A}, \xi _{A})$ is a picture fuzzy Lie ideal (sub-algebra) of a Lie algebra *L*, the following axioms then hold true:

1. $$\zeta _{A}(0) \ge \zeta _{A}(v_{1})$$, $$\eta _{A}(0) \ge \eta _{A}(v_{1})$$, $$\xi _{A}(0) \le \xi _{A}(v_{1});$$
2. $$\zeta _{A}([v_{1},v_{2}])= \zeta _{A}(-[v_{1},v_{2}])= \zeta _{A}([v_{2},v_{1}]),$$ $$\eta _{A}([v_{1},v_{2}])= \eta _{A}(-[v_{1},v_{2}])= \eta _{A}([v_{2},v_{1}],$$ $$\xi _{A}([v_{1},v_{2}])= \xi _{A}(-[v_{1},v_{2}])= \xi _{A}([v_{2},v_{1}]$$.

### Theorem 2

If all non-empty level subsets $U(\zeta _{P}, \varepsilon ),\; U(\eta _{P}, \varepsilon )$ and $L(\xi _{P}, \varepsilon )$ of $P=(\zeta _{P}, \eta _{P}, \xi _{P})$ are ideals in *L* (Lie algebra), this implies that *P*, a picture fuzzy set of *L*, is a picture fuzzy Lie ideal of *L*.

### Proof

Consider an $\varepsilon \in [0,1]$ such that $U(\zeta _{P}, \varepsilon ),\; U(\eta _{P}, \varepsilon )$ and $L(\xi _{P}, \varepsilon )$ are the ideals of *L*. Suppose $\zeta _{P}(u_{1}+u_{2})< \min \{\zeta _{P}(u_{1}), \zeta _{P}(u_{2})\} \; \forall \;u_{1},u_{2} \in U(\zeta _{P}, \varepsilon )$. Then choose an $\varepsilon \in [0,1]$ such that

$$\begin{aligned} \zeta _{P}(u_{1}+u_{2})< \varepsilon < \min \{\zeta _{P}(u_{1}), \zeta _{P}(u_{2})\}. \end{aligned}$$

It implies $\zeta _{P}(u_{1}+u_{2}) \notin U(\zeta _{P}, \varepsilon )$ (a contradiction). Thus

$$\begin{aligned} \zeta _{P}(u_{1}+u_{2}) \ge \min \{\zeta _{P}(u_{1}), \zeta _{P}(u_{2})\}. \end{aligned}$$

Similarly, for $\zeta _{P}(cu_{1}) \ge \zeta _{P}(u_{1})$ and $\zeta _{P}([u_{1}, u_{2}]) \ge \max \{\zeta _{P}(u_{1}), \zeta _{P}(u_{2})\}$. The proof for the right and left ideals is similar. $\square$

### Theorem 3

Consider a Lie algebra *L* and its picture fuzzy Lie ideal $A=(\zeta _{A},\eta _{A}, \xi _{A})$. It ensures that $\forall \; \varepsilon \in \zeta _{A}(L) \cap \eta _{A}(L) \cap \xi _{A}(L) \; \subseteq [0,1]$ level subsets, $U(\zeta _{A},\varepsilon ), \; U(\eta _{A},\varepsilon )$, and $L(\xi _{A}, \varepsilon )$ are the ideals in *L*, where $\zeta _{A}(L),\; \eta _{A}(L)$, and $\xi _{A}(L)$ are the respective collections of the values of $\zeta _{A}, \; \eta _{A}$ and $\xi _{A}$.

### Proof

The proof proceeds with proving that $u_{1}+u_{2}, \; cu_{1},\; [u_{1},u_{2}] \in U(\zeta _{A}, \varepsilon ), U(\eta _{A},\varepsilon )$ and $L(\xi _{A}, \varepsilon ), \; \forall \; u_{1},u_{2} \in X$ and $c \in {\mathbb {F}}$. Consider a picture fuzzy Lie ideal *A* of *L*, and $u_{1},u_{2} \in U(\zeta _{A}, \varepsilon )$, i.e., $\zeta _{A}(u_{1}) \ge \varepsilon$ and $\zeta _{A}(u_{2}) \ge \varepsilon$. We have the following two cases.

Case 1 ($$\zeta _{A}(u_{1}) < \zeta _{A}(u_{2})$$):

$$\begin{aligned} \zeta _{A}(u_{1}+u_{2}) \ge \min \{\zeta _{A}(u_{1}), \zeta _{A}(u_{2})\}= \zeta _{A}(u_{1}) \ge \varepsilon . \end{aligned}$$

Similarly,

$$\begin{aligned} \zeta _{A}(cu_{1}) \ge \varepsilon \text { and } \zeta _{A}([u_{1},u_{2}]) \ge \varepsilon . \end{aligned}$$

Similarly, for Case 2($\zeta _{A}(u_{2}) < \zeta _{A}(u_{1})$), we have the same result. Thus $u_{1}+u_{2}, \;cu_{1}$ and $[u_{1},u_{2}] \in U(\zeta _{A}, \varepsilon )$ and $U(\zeta _{A}, \varepsilon )$ is an ideal in *L*. $U(\eta _{A}, \varepsilon )$ and $L(\xi _A, \varepsilon )$ can be proved as picture fuzzy Lie ideals in a similar manner. This result is also valid for right and left ideals. $\square$

As a consequence of Theorems 2 and 3, we derive the following result.

### Corollary 1

A picture fuzzy set $P= (\zeta _{P}, \eta _{P}, \xi _{P})$ of *L* (a Lie algebra) is a picture fuzzy right (left/two-sided) Lie ideal (sub-algebra) iff $\forall \; (\varepsilon , \varsigma , \tau ) \in \zeta _{P}(L)\times \eta _{P}(L)\times \xi _{P}(L)$, ${\mathcal {P}}^{(\varepsilon , \varsigma , \tau )}=U(\zeta _{P}, \varepsilon ) \cap U(\eta _{P}, \varsigma ) \cap L(\xi _{P}, \tau )$ is a Lie ideal (sub-algebra) of *L*, where $\varepsilon + \varsigma + \tau \le 1$.

### Proposition 4

Consider a Lie algebra *L* and a collection of its picture fuzzy right (left/two-sided) Lie ideals $\{K_{i}: i \in I\}$, then:

$$\begin{aligned} \cap K_{i}=(\wedge \zeta _{K_{i}},\wedge \eta _{K_{i}},\vee _{K_{i}}) \end{aligned}$$

is also a picture fuzzy right(left/two-sided) Lie ideal of *L*, where

$$\begin{aligned} \wedge \zeta _{K_{i}}(x)& =  \inf \{\zeta _{K_{i}}(x) :\; i \in I, x \in L \}\\ \wedge \eta _{K_{i}}(x)& =  \inf \{\eta _{K_{i}}(x) :\; i \in I, x \in L \}\\ \vee \xi _{K_{i}}(x)& =  \sup \{\xi _{K_{i}}(x) :\; i \in I, x \in L \}. \end{aligned}$$

### Remark 2

1. The union of two picture fuzzy Lie ideals may or may not be a picture fuzzy Lie ideal.
2. The sum of two picture fuzzy Lie ideals is a picture fuzzy Lie ideal, too.

### Proposition 5

Consider a picture fuzzy Lie ideal (sub-algebra) $J_{k}$ of *L* (Lie algebra) $\forall \; k=1,2, \ldots ,i,i+1, \ldots$, so that $J_{k}$ forms the chain below

$$\begin{aligned} \cdots \subseteq J_{i} \subseteq J_{i+1} \subseteq \cdots . \end{aligned}$$

Then,

$$\begin{aligned} J_{i+1}/J_{i}: L/J_{i_{t}} \longrightarrow [0,1], \end{aligned}$$

defined as $J_{i+1}/J_{i}(\ell +J_{i_{t}})=J_{i+1}(\ell )$, where $\ell \in L$ and $J_{i}(0)=t$ is also a picture fuzzy Lie ideal (sub-algebra).

### Picture Fuzzy Lie Homomorphism

We are already familiar with the Lie homomorphism. Now we have its natural extension in picture fuzzy Lie algebra.

#### Definition 14

Let $J^{L}$ and $J^{L_{0}}$ be the collections of the picture fuzzy sets of Lie algebras *L* and $L_{0}$ respectively. A picture fuzzy Lie homomorphism $\omega \,: J^{L} \longrightarrow J^{L_{0}}$ is defined as:

$$\begin{aligned} \omega (\zeta _{A})(v)&= \left\{ \begin{array}{c c} \sup _{u \in \omega ^{-1}(v)}\{\zeta _{A}(u)\}, &{} \hbox { if } \omega ^{-1}(v) \ne \phi ; \\ 0, &{} \hbox { if } \omega ^{-1}(v) = \phi . \end{array} \right. \\ \omega (\eta _{A})(v)&= \left\{ \begin{array}{c c} \sup _{u \in \omega ^{-1}(v)}\{\eta _{A}(u)\}, &{} \hbox { if } \omega ^{-1}(v) \ne \phi ; \\ 0, &{} \hbox { if } \omega ^{-1}(v) = \phi . \end{array} \right. \\ \omega (\xi _{A})(v)&= \left\{ \begin{array}{c c} \inf _{u \in \omega ^{-1}(v)}\{\xi _{A}(u)\}, &{} \hbox { if } \omega ^{-1}(v) \ne \phi ; \\ 1, &{} \hbox { if } \omega ^{-1}(v) = \phi . \end{array} \right. \end{aligned}$$

$$\forall A= (\zeta _{A}, \eta _{A}, \xi _{A}) \in J^{L}$$ and $$v \in L_{0}$$.

#### Definition 15

Consider a homomorphism $\vartheta$ between two Lie algebras *L* and $L_{0}$. Let *A* and *B* be the picture fuzzy sets in *L* and $L_{0}$ respectively. Then it is said that under $\vartheta$, *B* has the pre-image, which is a picture fuzzy set formalized as

$$\begin{aligned} \vartheta ^{-1}(B)= (\vartheta ^{-1}(\zeta _{B}), \vartheta ^{-1}(\eta _{B}), \vartheta ^{-1}(\xi _{B}))= B(\vartheta ). \end{aligned}$$

#### Theorem 6

Considering a monomorphism $\vartheta :L \longrightarrow L_{0}$ of a Lie algebras, then:

1. A picture fuzzy Lie sub-algebra (right/left/two-sided Lie ideal) always has a picture fuzzy Lie sub-algebra (right/left/two-sided Lie ideal) as its image.
2. The pre-image of a picture fuzzy right (left/two-sided) Lie ideal (picture fuzzy Lie sub-algebra) is also a picture fuzzy right (left/two-sided) Lie ideal (picture fuzzy Lie sub-algebra).

#### Proof

1. Consider *u* and *v* in $L_{0}$. Then
  $$\begin{aligned} \{c :\; c \in \vartheta ^{-1}(u+v)\} \supseteq \{c_{1}+c_{2}:\; c_{1} \in \vartheta ^{-1}(u) \; and\; c_{2} \in \vartheta ^{-1}(v)\}. \end{aligned}$$
  Now,
  $$\begin{aligned}&\vartheta (\zeta _{A})(u+v) = \sup \{\zeta _{A}(c)\;:\; c \in \vartheta ^{-1}(u+v)\}\\&\ge \sup \{\zeta _{A}(c_{1}+c_{2})\;:\; c_{1}\in \vartheta ^{-1}(u)\; and\; c_{2} \in \vartheta ^{-1}(v)\}\\&\ge \sup \{\min \{\zeta _{A}(c_{1}), \zeta _{A}(c_{2})\}\;:\; c_{1}\in \vartheta ^{-1}(u)\; and\; c_{2} \in \vartheta ^{-1}(v)\}\\&=\min \{\sup \{\zeta _{A}(c_{1})\;:\; c_{1} \in \vartheta ^{-1}(u)\}, \sup \{\zeta _{A}(c_{2}) \;:\; c_{2}\in \vartheta ^{-1}(v)\}\}\\&=\min \{\vartheta (\zeta _{A})(u), \vartheta (\zeta _{A})(v)\}. \end{aligned}$$
  Similarly by considering
  $$\begin{aligned} \{c :\; c \in \vartheta ^{-1}([u,v])\} \supseteq \{[c_{1},c_{2}]:\; c_{1} \in \vartheta ^{-1}(u) \; and\; c_{2}\in \vartheta ^{-1}(v)\}, \end{aligned}$$
  we can prove that $\vartheta (\zeta _{A})([u, v]) \ge \min \{\vartheta (\zeta _{A})(u), \vartheta (\zeta _{A})(v)\}$ and again $\vartheta (\zeta _{A})(\alpha u) \ge \vartheta (\zeta _{A})(u)$. The same can be proved for $\eta _{A}$ and $\xi _{A}$, and thus $\vartheta (A)$ becomes a picture fuzzy Lie sub-algebra of $L_{0}$.
2. Let $Q=(\zeta _{Q}, \eta _{Q}, \xi _{Q})$ be a picture fuzzy right (left/two-sided) ideal of $L_{0}$. Then its pre-image is $\vartheta ^{-1}(Q)= (\vartheta ^{-1}(\zeta _{Q}), \vartheta ^{-1}(\eta _{Q}), \vartheta ^{-1}(\xi _{Q}))$. Let $u_{1},u_{2} \in L$ and $c \in {\mathbb {F}}$, then the proof follows from Definitions 13 and 15.

$$\square $$

#### Definition 16

For a picture fuzzy Lie ideal(sub-algebra) $A=(\zeta _{A}, \eta _{A}, \xi _{A})$ in *L*, $x \in L$ and *A* determines a picture fuzzy coset $A_u^*$ with degrees defined as follows:

$$\begin{aligned} \zeta _{A^*}(c) = \zeta _{A}(c-u), \quad \eta _{A^*}(c) = \eta _{A}(c-u), \quad \xi _{A^*}(c) = \xi _{A}(c-u),\quad \forall \; c \in L. \end{aligned}$$

Moreover, the set *L*/*A* of all the picture fuzzy cosets of *A* in *L* is a Lie algebra under the following operations

$$\begin{aligned} A_{u_{1}}^*+ A_{u_{2}}^*&= A_{u_{1}+u_{2}}^*&\forall \, u_{1},\; u_{2}&\in L\\ d A_{u_{1}}^*&= A_{du_{1}}^*&\forall \, d \in {\mathbb {F}}; \; u_{1}&\in L\\ [A_{u_{1}}^*, A_{u_{2}}^*]&= A_{[u_{1}, u_{2}]}^*&\forall \, u_{1},\; u_{2}&\in L. \end{aligned}$$

#### Theorem 7

For a Lie algebra *L* and its picture fuzzy Lie ideal *A*. The map $\vartheta : L \longrightarrow L/A$ defined by $\vartheta (u) = A_{u}^{*}\; \forall \; u \in L$ is a Lie homomorphism with kernel ${\mathcal {A}}^{(0, 0, 1)}.$

#### Proof

It is evident that $\vartheta$ is a Lie homomorphism. We ought to show that $Ker(\vartheta ) ={\mathcal {A}}^{\varepsilon , \varsigma , \tau }$. Let $A(r)=A(0)$ and $a \in L$, then $A(a) \subseteq A(0)= A(r)$, and

$$\begin{aligned} \zeta _{A}(a)&= \zeta _{A}(a+r-r)\\&\ge \min \{\zeta _{A}(a-r), \zeta _{A}(r)\}\\&= \zeta _{A}(a-r)\\&\ge \min \{\zeta _{A}(a), \zeta _{A}(r)\} = \zeta _{A}(a)\\ \Rightarrow \zeta _{A}(a)&= \zeta _{A}(a-r) \end{aligned}$$

We can prove the same for $\eta _{A}$ and $\xi _{A}$. Thus $A(a)= A(a-r)$, i.e., $A^{*}_{0} = A^{*}_{r}.$ Also $A_{0}^{*}= A_{r}^{*} \Rightarrow A(0)=A(r)$. Hence,

$$\begin{aligned} Ker(\vartheta )&=\{u\in L \, : \, \vartheta (u)= A_0^*\}\\&=\{u\in L \, : \, A_u^*= A_0^*\}\\&=\{u\in L \, : \, A(u)= A(0)\}\\&=\{u\in L \, : \,\zeta _A(u)=0,\, \eta _A(u)=0,\, \xi _A(u)=1\}\\&={\mathcal {A}}^{(0, 0, 1)}. \end{aligned}$$

$$\square $$

#### Theorem 8

(First Isomorphism Theorem) Consider an epimorphism $\vartheta : L \longrightarrow L_{0}$, where *L* and $L_{0}$ are Lie algebras. Let $Q=(\zeta _{Q}, \eta _{Q}, \xi _{Q})$ be a picture fuzzy Lie ideal(sub-algebra) in $L_{0}$. Then $L/\vartheta ^{-1}(Q) \cong L_{0}/Q.$

#### Proof

Define $$ \sigma : L/\vartheta ^{-1}(Q) \longrightarrow L_{0}/Q$$ as

$$\begin{aligned} \sigma (\vartheta ^{-1}(\zeta _{Q}(v)))&= \zeta _{Q}(\vartheta (v)),\\ \sigma (\vartheta ^{-1}(\eta _{Q}(v)))&= \eta _{Q}(\vartheta (v)),\\ \sigma (\vartheta ^{-1}(\xi _{Q}(v)))&= \xi _{Q}(\vartheta (v)),\quad \forall \;v \in L, \; Q \in J^{L_{0}}. \end{aligned}$$

Then $\sigma$ is an isomorphism. $\square$

#### Theorem 9

(Second Isomorphism Theorem) Let *L* be a Lie algebra and $P=(\zeta _{P}, \beta _{P}, \xi _{P})$ and $Q=(\zeta _{Q}, \eta _{Q}, \xi _{Q})$ be its picture fuzzy Lie ideals (sub-algebras), with $P(0) = Q(0)$. Then

$$\begin{aligned} (L_{P} +L_{Q})/{Q} \cong {L_{P}}/{(P\cap Q)}. \end{aligned}$$

#### Proof

Clearly, $L_{P} + L_{Q}$ and $L_{P}/(P \cap Q)$ are picture fuzzy Lie ideals (sub-algebras) in *L*. For any $x \; \in (L_{P}+L_{Q}),\; x=y+z$, where $y\; \in L_{P}$ and $z\; \in L_{Q}$. Let *P* and *Q* have a congruence relation $\sim$ on *L*, defined as $y \sim u \Leftrightarrow P(y-u)= P(0)$ (for *P*) and $Q(y-u)=Q(0)$ (for *Q*). Define $\sigma :L_{P}+L_{Q}/Q \longrightarrow L_{P}/P \cap Q$ as $\sigma (Q(x))= \sigma (Q(y+z))= (P \cap Q)(y)$. Then $\sigma$ fulfills all the requirements for an isomorphism. $\square$

#### Theorem 10

(Third Isomorphism Theorem) Let $P=(\zeta _{P}, \eta _{P}, \xi _{P})$ and $Q=(\zeta _{Q}, \eta _{Q}, \xi _{Q})$ be two picture fuzzy sets of a Lie algebra *L* with $P \subseteq Q$ and $P(0) = Q(0)$. Then,

$$\begin{aligned} L/P/L_{Q}/P \cong L/P. \end{aligned}$$

#### Proof

Clearly, $L/P/L_{Q}/P$ is a picture fuzzy Lie ideal(sub-algebra) over *L*. Define

$$\begin{aligned} \sigma :L/P/L_{Q}/P \longrightarrow L/Q\; as \; \sigma (P(x)+L_{Q}/P)= Q(x), \; \forall x\; \in L. \end{aligned}$$

Then the proof is straightforward. $\square$

## Nilpotency and Solvability of Picture Fuzzy Lie Ideals

So far, we have studied the nilpotent and solvable Lie algebras. Using the derived series of a picture fuzzy Lie ideal *S* in a Lie algebra:

$$\begin{aligned} S^{(0)} \supseteq S^{(1)} \supseteq \cdots \supseteq S^{(k)} \supseteq \ldots , \end{aligned}$$

we define its solvable Lie algebra from the lower central series as follows:

$$\begin{aligned} N^{0} \supseteq N^{1} \supseteq N^{2} \supseteq \cdots \supseteq N^{k} \supseteq \ldots , \end{aligned}$$

The nilpotent Lie algebra is constructed. In the Lie algebra, solvable and nilpotent Lie algebras are of great importance as they appear abundantly. For example, the collection of strictly upper triangular matrices over the field ${\mathbb {F}}$ is nilpotent, and that of the upper triangular matrices is solvable. Let us now define nilpotent and solvable Lie algebras over a picture fuzzy set.

### Definition 17

For a picture fuzzy Lie ideal *S* in *L* (Lie algebra), its derived series is written as

$$\begin{aligned} S^{(0)} \supseteq S^{(1)} \supseteq \cdots \supseteq S^{(k)} \supseteq \ldots , \end{aligned}$$

and we say that $S^{(k)}$ is the *k*th derived picture fuzzy Lie ideal of the Lie algebra, where

$$\begin{aligned} S^{(0)}= S,\; S^{(1)}=[S^{(0)}, S^{(0)}],\ldots ,S^{(k)}=[S^{(k-1)},S^{(k-1)}]. \end{aligned}$$

If $\exists \, \aleph \in {\mathbb {Z}}^{+}$ such that $S^{(\aleph )}=0_{\sim }$, then *S* is termed as a solvable picture fuzzy Lie ideal of *L*. Similarly, in the lower central series of *S* i.e.

$$\begin{aligned} S^{0} \supseteq S^{1} \supseteq S^{2} \supseteq \cdots \supseteq S^{k} \supseteq \ldots , \end{aligned}$$

when $S^{\aleph }=0_{\sim }=(0,0,1)$ for some $\aleph \in {\mathbb {Z}}^{+}$, then *S* is known as nilpotent picture fuzzy Lie ideal. Here

$$\begin{aligned} S^{0}=S, \; S^{1}= [S, S^{0}], \ldots ,S^{k}=[S, S^{k-1}]. \end{aligned}$$

### Theorem 11

Let *S* be a solvable/nilpotent picture fuzzy Lie ideal of a Lie algebra *L*. Then

1. Each picture fuzzy set $R \subseteq S$ is also a solvable/nilpotent picture fuzzy Lie ideal of a Lie algebra;
2. Let *R* be a picture fuzzy subset of *S*, then *S*/*R* is also solvable/nilpotent.

### Proof

1. It is known that,
  $$\begin{aligned} R^{(1)} = [R,R] \subseteq [S, S] = S^{(1)}. \end{aligned}$$
  Similarly, $R^{(k)} \subseteq S^{(k)}$ for any *k*. Therefore, we may induce that
  $$\begin{aligned} R^{(k)} \subseteq S^{(k)} = 0_{\sim }. \end{aligned}$$
2. We define *S*/*R* as $S/R(u+R_{t}) =S(u)\; \forall \; u \in L$ and $t= R(0)$ through the map $S/R:L/R_{t} \longrightarrow [0,1].$ Then the proof follows the definition of $S^{(1)}=[S, S]$.

$$\square $$

### Proposition 12

Consider a picture fuzzy subset *R* of a picture fuzzy Lie ideal *S* in a Lie algebra *L*. If *S*/*R* is a solvable picture fuzzy Lie ideal of *L*, then it turns out that *S* is solvable as well.

### Theorem 13

In the homomorphisms, a solvable picture fuzzy Lie ideal only has a solvable picture fuzzy Lie ideal as its image.

### Proof

We know that $\vartheta (S^{(k)}) \subseteq (\vartheta (S))^{(k)}$. Let there be a Lie homomorphism $\vartheta$ from *L* to $L_{0}$ and *S* be the picture fuzzy Lie ideal in *L*. Through the induction on

$$\begin{aligned} \vartheta (S^{(k)}) \supseteq [\vartheta (S)]^{(k)}, \qquad k \in {\mathbb {Z}}^{+} \end{aligned}$$

our result can be very easily proved. Starting from $k = 1$,

$$\begin{aligned} \vartheta ([S,S]) \supseteq [\vartheta (S), \vartheta (S)]. \end{aligned}$$

Let $$ v \in L_{0}$$. Then

$$\begin{aligned} \vartheta ([\zeta _{S}, \zeta _{S}])(v) =\sup \{ [\zeta _{S}, \zeta _{S}](u):\; \vartheta (u)=v\}. \end{aligned}$$

Now following the definition of the picture fuzzy Lie product, we can prove that

$$\begin{aligned} \vartheta ([\zeta _{S}, \zeta _{S}]) \ge [\vartheta (\zeta _{S}), \vartheta (\zeta _{S})](v). \end{aligned}$$

The same can be proved for $\eta _{S}$ and $\xi _{S}$. Thus

$$\begin{aligned} \vartheta ([S,S]) \supseteq [\vartheta (S), \vartheta (S)]. \end{aligned}$$

Now for $k \ge 1$, we get

$$\begin{aligned} \vartheta (S^{(k)})&=\vartheta ([S^{(k-1)}, S^{(k-1)}]) \supseteq [\vartheta (S^{(k-1)}), \vartheta (S^{(k-1)})]\\&\supseteq [(\vartheta (S))^{(k-1)}, (\vartheta (S))^{(k-1)}]= (\vartheta (S))^{(k)}\\ \Rightarrow \qquad \qquad \qquad \vartheta (S^{(k)})&\supseteq (\vartheta (S))^{(k)}, \end{aligned}$$

but $$\vartheta (S^{(k)}) \subseteq (\vartheta (S))^{(k)}.$$ Thus

$$\begin{aligned} \vartheta (S^{(k)})= (\vartheta (S))^{(k)}. \end{aligned}$$

Since, $S^{(k)}= 0_{\sim }$, we may get $\vartheta (S^{(k)})=\vartheta (0_{\sim })= (\vartheta (S))^{(k)}$. $\square$

Following a similar argument we get the result for a nilpotent picture fuzzy Lie algebra stated as follows:

### Theorem 14

Let $\vartheta$ be a homomorphism. Then a nilpotent picture fuzzy Lie ideal only has a nilpotent picture fuzzy Lie ideal as its image.

$$a_1, a_2, a_3, b_1, b_2, b_3, c_1, c_2, c_3$$

## Conclusion

As discussed earlier, the picture fuzzy set is a generalization of fuzzy and intuitionistic fuzzy sets. The notion of picture fuzzy Lie algebra presented in this paper may provide a scheme and open new doors for scientific discoveries and their outcomes. The utmost importance of Lie algebra was a motivation to study Lie sub-algebra and ideal under picture fuzzy environment, which has been successfully defined. Picture fuzzy Lie homomorphism is then laid out using the definitions of Lie homomorphism. By the same token, the sheer value of nilpotent and solvable Lie algebras was analyzed, and thus discussed over picture fuzzy sets.

It is a matter of great interest and a task to apply this logical framework to the problems in quantum mechanics and physics. Further, the discussion can be concluded by the fact that the Lie algebra has wide application in physics, continuum mechanics, and cosmology, so any advancement in the field of the picture fuzzy Lie algebra will ultimately benefit in these areas.

## Data Availability

No data were used to support this study.
